# Mapping gender networks of smartphone addiction and academic procrastination: a network analysis study

**DOI:** 10.3389/fpsyg.2025.1557684

**Published:** 2025-06-18

**Authors:** Lu Song, Zhilin Liu, Yujia Yang, Shuangshuang Yuan

**Affiliations:** ^1^School of Education Science, Liupanshui Normal University, Liupanshui, China; ^2^Department of Education, Nanchang Institute of Science and Technology, Nangchang, Jiangxi, China; ^3^College of Music, Luoyang Normal University, Luoyang, China; ^4^Department of Life Science, Changzhi University, Changzhi, China

**Keywords:** gender studies, adult learning, pedagogical issues, smartphone addiction, academic procrastination

## Abstract

**Background:**

Smartphone addiction (SA) and academic procrastination (AP) are two of the educational challenges encountered by many higher education students today that have led to a series of adverse effects on their well-being.

**Aims, sample, and methods:**

Previous studies have reported inconsistent findings regarding gender differences in SA and AP, and limited attention has been paid to gender differences in the interaction between SA and AP. To address this gap, the present study employs network analysis to investigate gender differences in the SA, AP, and SA-AP interaction networks among higher education students. A total of 438 students from four higher education institutions in China participated in this study.

**Results:**

The findings indicate that there are more differences than similarities in the SA and AP networks between male and female students. In addition, within the SA-AP interaction network, the core feature of the male network is academic procrastination, reflecting deficiencies in time management and self-regulation. In contrast, the core feature of the female network is smartphone addiction, which highlights a strong dependence on immediate social feedback.

**Conclusion:**

This study represents the first attempt to investigate gender differences in SA and AP through the lens of network analysis. The findings reveal the complexity of gender differences in behavioral patterns and psychological mechanisms, moving beyond the limitations of previous research that primarily focused on mean-level differences. This study deepens the understanding of SA and AP and provides both theoretical support and practical guidance for the development and implementation of effective intervention strategies to address SA and AP among students.

## 1 Introduction

The number of smartphone users is continuously increasing. For example, China, one of the world’s largest economies, has 986 million smartphone users, while in the United States, more than 90% of 18-year-olds own smartphones (Rideout and Robb, 2019). Notably, in 2021, the global number of smartphone users was projected to surpass 6.37 billion (Statista, 2021). The increasing trend of smartphone users can be attributed to the instant gratification for social, entertainment, and information acquisition needs of users, especially in times of facing negative emotions such as stress and anxiety (Cho et al., 2017; Monacis et al., 2020). This could lead to the onset of smartphone addiction (SA), including but not limited to the experience of excessive smartphone use and the feeling of anxiety when separated from smartphones (Akinci, 2021). Noticeably, SA could have damaging impacts on students, such as an undermined academic performance with declining grades and academic procrastination and affecting their well-being such as afflicted with carpal tunnel syndrome, withdrawal, and anxiety (Albursan et al., 2022; Buctot et al., 2020). The relationship between SA and AP has garnered widespread academic attention (Albursan et al., 2022; Li et al., 2020; Rozgonjuk et al., 2018). Researchers have found a connection between SA and AP (Akinci, 2021; Saad and Khalifa, 2020). Nevertheless, there is a lack of research on gender differences in this relationship. As a result, there may not be sufficient guidance for educators and researchers to develop and implement effective SA and AP interventions. Moreover, the inconsistent findings from previous studies do not deliver convincing evidence on gender differences in SA and AP because most of these studies have examined SA and AP as separate constructs, rather than as a whole or as related (Gezgin, 2021; Üztemur and Dinç, 2023; Zhou, 2020). This underscores the urgent need to investigate gender differences in SA and AP. As a response, this study focuses on gender differences in SA and AP among higher education students to address this gap in the literature.

### 1.1 Gender differences in SA

Tu et al. (2023) assert that SA is characterized by the excessive desire to use smartphones, which impairs psychological and social functioning. SA is prevalent and commonly found among higher education students (Gao et al., 2018). Empirical studies indicate approximately 62.6% of university students in the Philippines (Buctot et al., 2020), 70% of South Korean university students (Kwak et al., 2018), and 40% of university students in China (Zhang et al., 2022) struggle with SA. Moreover, SA is typically associated with a range of psychological and physiological symptoms such as compulsive behaviors, withdrawal, anxiety, difficulty in concentrating, migraine headaches, back pain, and loss of sound sleep (Albursan et al., 2022; Yildiz Durak, 2019), all of which can jeopardize the academic performance and overall well-being of students. Studies have pointed out that the formation of SA among students is rather complicated and encompasses multiple factors such as maladjustment, personality traits, stress, academic self-efficacy, conflicting family and peer relationships, and social maladjustment (Gezgin, 2021; Kwak et al., 2018; Peng et al., 2022; Yang et al., 2019; Yang et al., 2020; Zhang et al., 2022).

Gender differences are currently one of the most controversial topics in SA research (Gezgin, 2021; Üztemur and Dinç, 2023). Some studies indicate that males have higher SA than females (e.g., Albursan et al., 2019; Buctot et al., 2020; Qudah et al., 2019), while other studies suggest that female has higher SA than boys (e.g., Park et al., 2019; Tu et al., 2023; Zhao et al., 2021). Interestingly, some studies have even suggested that there is no difference in SA between genders (e.g., Gezgin, 2021; Przepiorka et al., 2021). These conflicting findings may stem from various factors. For instance, Su et al. (2019) pointed out that the gender differences in SA could be attributed to the differences in economic and internet availability of different countries. This is because stronger economies are often associated with higher internet penetration rates, and greater internet availability may increase the likelihood of SA. Additionally, economic development is closely linked to gender equality, which encompasses digital inclusion. As the GDP per capita and internet penetration rate of a country increase, gender differences in internet-related addictive behaviors, including SA also tend to decrease (Woetzel et al., 2015). Alternatively, Gezgin (2021) explained the inconsistent results based on the habits and purposes of smartphone use. He argues that males tend to use smartphones more for gaming and thus score higher on the measurement scale. Females, on the other hand, are prone to use multimedia applications and social networking services more, thus, scoring higher on these dimensions (Chen et al., 2017). However, many existing studies have not considered the genders discrepancies in usage patterns -, which also contributes to the inconsistent results. Therefore, it is important to investigate further gender differences in SA to foster clarity and contribute to the field of knowledge.

### 1.2 Gender differences in AP

Academic Procrastination (AP) refers to the habitual postponement of academic tasks, despite the potential for negative outcomes (Simpson and Pychyl, 2009; Steel, 2007) such as self-blame, depression, and poor academic performance (Ashraf et al., 2023). AP is very common among higher education students (Steel, 2007; Üztemur and Dinç, 2023), with empirical studies indicating that approximately 70% of students frequently experience AP (Rabin et al., 2011), especially during thesis writing, exam preparation, and assignment submission (Onwuegbuzie and Jiao, 2000). The causes of AP are rather complex and are often associated with factors such as low academic self-efficacy, poor self-regulation and academic pressure (Ashraf et al., 2023; Grunschel et al., 2013; Yang et al., 2020; Zhou, 2020).

Similar to SA, the findings on gender differences in AP from previous studies are also conflicting (Üztemur and Dinç, 2023; Zhou, 2020). Some studies have reported that males exhibit higher AP compared to females (Albursan et al., 2022; Balkis and Duru, 2017), while others indicate higher AP among females (Rodarte-Luna and Sherry, 2008). Moreover, there is also evidence revealing that there is no difference in AP between males and females (Argiropoulou et al., 2020). The conflicting findings may be attributed to the differences in academic pressures that are experienced by the two genders in different cultures. For example, in collectivist cultures that emphasize collective interests, females may face academic pressure primarily from family supervision and demands (Peleg et al., 2016), while males experience pressure from both family and societal expectations, including future employment prospects (Xie, 2021). In contrast, in individualist cultures that stress individual rights, academic pressure on both males and females is more likely to come from the gap between their personal academic expectations and reality (Özer et al., 2009). Overall, AP is one of the academic issues that is faced by the majority of higher education students. It is difficult to deliver appropriate and effective interventions without understanding the gender differences. Therefore, further investigation on this matter is crucial to address the specific needs of both male and female students.

### 1.3 The interaction relation between SA and AP

A large body of empirical research indicates there is a significant relationship between SA and AP (Akinci, 2021; Albursan et al., 2022; Li et al., 2020). The findings from the literature support that the association between these two constructs is mutual. In cases where AP positively predicts SA, students perceive pressure from academic tasks and consider the given tasks to be boring and too complex, leading them to procrastinate (Reinecke et al., 2018; Rozgonjuk et al., 2018). In such situations, smartphones with high accessibility to entertainment and social networks serve as emotional regulators, allowing students to avoid or escape from academic tasks by offering instant gratification (Üztemur and Dinç, 2023; Yang et al., 2020). Similarly, SA also positively predicts AP by reducing academic motivation, self-regulation, and attention in classes, thereby inducing procrastination behavior (Geng et al., 2018; Rozgonjuk et al., 2018; Yang et al., 2019). The mutual interaction between SA and AP has drawn scholars’ attention to further investigate the mediating and moderating variables influencing SA and AP in order to address their negative effects on students (Li et al., 2020; Üztemur and Dinç, 2023; Yang et al., 2020). For example, Liu et al. (2022) found that self-efficacy, academic pressure, learning strategies, and time management play mediating roles, while Üztemur and Dinç (2023) found that self-control acts as a moderator. These studies not only have theoretical significance for a deeper understanding of the interaction between SA and AP but also have practical significance for mitigating the negative impact of SA and AP on higher education students.

Despite the identified significant relationship between SA and AP, previous studies have overlooked the role of gender differences, as gender has not been considered a factor in these prior studies. Therefore, network analysis offers a new perspective on the mutual influence of these variables, presenting them in terms of network structure, connectivity, and interactions. This approach provides new insights into the complex dynamic interactions between SA and AP across gender. This study seeks to investigate the gender difference in the network that is formed by the interaction between SA and AP and interpret the findings from the core features and network. This study not only examines gender differences in SA and AP networks separately but also the interactive relation between SA and AP. The network analysis method is appropriate to be employed in this study as it enables the examination of multiple variables in the interactive networks. This approach is also capable of identifying the core features and the interactions between specific sets of features of the network, as well as enabling network comparison (Song et al., 2023; Yang et al., 2024). In light of this, this study adopts network analysis methods to construct networks of SA and AP for males and females respectively, and analyzes the differences between the two genders from the core features and the network.

### 1.4 Network analysis

Network analysis constructs a network model that includes nodes and edges based on variable items (Epskamp et al., 2018). The nodes signify the items in the variables, the edges signify the interactions among the items, and the edge weights signify the intensity of the correlation connecting two items (Borsboom and Cramer, 2013). Recently, network analysis has gained popularity in research aimed at exploring gender differences (Maccallum et al., 2021). Furthermore, network analysis can compare the differences between two or more networks. For instance, Liu et al. (2023) and Wang et al. (2022) employ network analysis to investigate gender differences in their studies. These recent studies suggest that network analysis aligns with the purpose of this study and is deemed appropriate. Moreover, network analysis identifies core features (or core items) within the network model. Core features would display many links with other features (Borsboom and Cramer, 2013), and stimulation of a core feature activates other components of the network.

Network comparison could evaluate if there are significant differences in network structure, global strength, and edge strength among multiple networks (van Borkulo et al., 2015). If the network structure is similar, it indicates that the relationships between items are comparable overall. Networks with greater global strength might exhibit more robust feedback loops between items, and the strength of edges could further reveal differences in the correlation between features (Benfer et al., 2018).

### 1.5 Present study

SA and AP are prevalent phenomena among higher education students, and they have various negative impacts on the students’ well-being. Considering the ongoing debates regarding gender differences in SA and AP, and the limited research on gender differences in the interaction between SA and AP, this study aims to address these gaps and controversies through the lens of network analysis. The findings of the study offer new insights into gender differences in SA and AP among higher education students by focusing on specific features and the network. In this regard, the study has formulated three research questions, which are as follows:

RQ1: Are there any differences in the AP networks between male and female higher education students?

RQ2: Are there any differences in the SA networks between male and female higher education students?

RQ3: Are there any differences in the interaction networks of SA-AP between male and female higher education students?

## 2 Methodology

### 2.1 Sampling

This study adopts a multi-stage sampling method. In the initial stage, a random number was assigned to each of the 34 provinces of China, and two of these numbers were randomly selected using the Online Random Number Generator website (Calculator.net, 2023). Similarly, in the second stage, two universities were randomly selected from each of the chosen provinces using the same website. In the third stage, stratified random sampling was utilized to determine the proportion of the sample needed at each selected university based on the calculated sample size. Lastly, simple random sampling using the Online Random Number Generator website (Calculator.net, 2023) was applied to select eligible participants from the established sample frame.

### 2.2 Participants

Considering that the first-year students had just started their courses and the fourth-year students were busy with internships, this study solely focused on the second and third-year students from 4 different universities in two provinces of China with a total population of 29,553. Subsequently, the researchers sent the survey link and informed consent form to the respective participants through email. Ultimately, 477 participants responded. However, 39 responses were deemed invalid due to arbitrary marking or outliers. Therefore, 438 responses were valid for data analysis. The mean age of the participants was 20.16 years old with a standard deviation of 0.83. Of these, 268 were females (61.2%) and 170 were males (38.8%).

### 2.3 Instruments

This study adopts the Smartphone Addiction Scale-Short Version (SAS-SV) developed by Kwon et al. (2013) to assess the SA of participants. The SAS-SV consists of 10 items (e.g., Missing planned work due to smartphone use) and has been widely employed to measure SA among higher education students. The scale is structured around six dimensions: daily-life disturbance, positive anticipation, withdrawal, cyberspace-oriented relationship, overuse, and tolerance, which capture different facets of smartphone addiction. Additionally, the SAS-SV is a validated scale that has been used in previous studies in China (Zhao et al., 2022), making it appropriate for this study. This study employs the Procrastination Scale that has been developed by Tuckman (1991) to measure AP among the participants. The scale comprises 16 items (e.g., I needlessly delay finishing jobs, even when they are important), which have demonstrated high internal consistency and have been used in prior Chinese studies (Li et al., 2020). The scale comprises two key dimensions: avoid unpleasant tasks and externalization of blame for unpleasant tasks. The Cronbach’s alpha coefficients for the scales in this study were 0.84 for SA and 0.89 for AP, indicating a reliable internal consistency.

### 2.4 Data collection

This study received approval from the academic ethics committee of the selected universities. The data for this study were gathered between January 12th and June 24th, 2023, using the online questionnaire platform Questionnaire Star.^1^ After receiving the list of students from the authorities and establishing the sample frame, an email containing the survey links and informed consent form was sent to the eligible participants. Moreover, participants received a survey introduction and instructions to review before completing the survey, ensuring they understood the study’s purpose and procedures. The process of sending invitations continued until sufficient responses were obtained for data analysis.

### 2.5 Data analysis

Prior to data analysis, this study assessed the normal distribution of the data using Skewness and Kurtosis. The Skewness values varied from −0.837 to 0.271, and the Kurtosis values ranged from −0.931 to 3.426; both sets of values fell within acceptable limits (Skewness < 3 and Kurtosis < 10). This study began with descriptive statistical analyses using SPSS version 23.0, followed by core feature analysis of the SA and AP conducted in R version 3.6.3 within RStudio version 1.2.5033.

#### 2.5.1 Network estimation

Following the guidelines set by Epskamp et al. (2018), the EBICglasso function from the *qgraph*, *v. 1.9.2* package was utilized to assess the network structure. This study estimated a Gaussian graphical model through a graphical lasso (i.e., glasso) combined with the extended Bayesian information criterion model. The GGM is applied to non-binary data. The thickness of the edge indicates an estimate of the bias correlation coefficient, while the color of the edge signifies the positive or negative correlation between the two items. In this study, network estimates were conducted separately for male and female samples.

#### 2.5.2 Estimation of centrality indicators

The centrality Plot function in the *qgraph*, *v. 1.9.2* package was used to calculate centrality metrics, which include strength and expected influence. A node with high strength has a stronger direct connection to other nodes, while a node with high expected influence signifies greater centrality and importance within the network.

#### 2.5.3 Network accuracy and stability

The *bootnet, v. 1.5* package was employed to evaluate the accuracy and stability of the network structure. On the one hand, the accuracy of edge weights was assessed by bootstrapping the 95% confidence intervals (CIs) for the edge weights. Narrow bootstrapped CIs indicated low sampling variability, suggesting that the estimated network was reliable. On the other hand, the stability of node centrality was assessed using case-dropping subset bootstrapping. The correlation stability coefficient (CS-coefficient) was applied to quantify this stability, with values above 0.25 considered acceptable and those exceeding 0.5 deemed excellent (Epskamp et al., 2018).

#### 2.5.4 Network comparison

The *NetworkComparisonTest, v. 2.2.1* package was used to identify differences between male and female networks. Three aspects were examined in this study: network structure invariance, global strength invariance, and edge strength invariance. The network structure invariance test assesses differences in the maximum edge strength within the network, while the global strength invariance test evaluates the overall strength difference across the network. Additionally, the edge strength invariance test focuses on differences between individual edges.

## 3 Results

### 3.1 Network of AP among male and female students

#### 3.1.1 Network estimation of AP among male and female students

**Figure 1:**
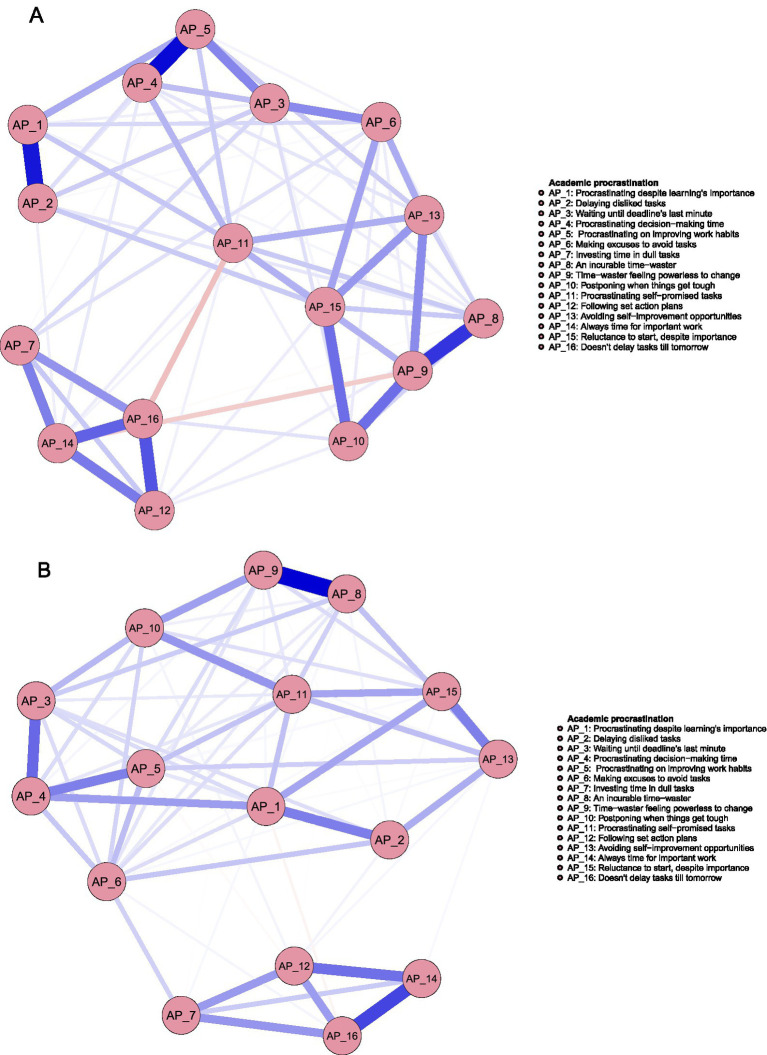
**(A)** Male. **(B)** Female.

The two networks produced a total of 120 edges (16*(16–1)/2), with 66 nonzero-weighted edges in males and 72 in females, as illustrated in Figures 1A,B.

In the visualized networks in Figures 1A,B, nodes correspond to specific AP features, while edges represent the relationships between them. The width of the edges reflects the strength of the partial correlations, with blue indicating positive correlations and red indicating negative ones. For a detailed explanation of the AP items, see Table 1.

**Table 1:** The network nodes of AP.

Features cluster		Item	Male sample (*N* = 170)		Female sample (*N* = 268)	
			*M*	*SD*	*M*	*SD*
AP 1	*Academic procrastination*	Procrastinating despite learning’s importance.	3.35	1.15	3.51	0.95
AP 2	*Academic procrastination*	Delaying disliked tasks.	3.23	1.19	3.30	0.96
AP 3	*Academic procrastination*	Waiting until deadline’s last minute.	3.64	1.09	3.82	0.90
AP 4	*Academic procrastination*	Procrastinating decision−making time.	3.43	1.19	3.66	0.94
AP 5	*Academic procrastination*	Procrastinating on improving work habits.	3.46	1.17	3.52	1.01
AP 6	*Academic procrastination*	Making excuses to avoid tasks.	3.51	1.16	3.65	0.97
AP 7	*Academic procrastination*	Investing time in dull tasks.	2.96	1.09	3.03	0.98
AP 8	*Academic procrastination*	An incurable time−waster.	3.51	1.18	3.68	1.01
AP 9	*Academic procrastination*	Time−waster feeling powerless to change.	3.48	1.16	3.68	1.02
AP 10	*Academic procrastination*	Postponing when things get tough.	3.55	1.11	3.63	0.98
AP 11	*Academic procrastination*	Procrastinating self−promised tasks.	3.55	1.10	3.68	0.95
AP 12	*Academic procrastination*	Following set action plans.	3.12	1.10	3.06	0.98
AP 13	*Academic procrastination*	Avoiding self−improvement opportunities.	3.4	1.09	3.56	0.97
AP 14	*Academic procrastination*	Always time for important work.	2.98	1.16	2.93	0.95
AP 15	*Academic procrastination*	Reluctance to start, despite importance.	3.47	1.06	3.56	1.02
AP 16	*Academic procrastination*	Does not delay tasks till tomorrow.	3.09	1.11	3.12	1.02

#### 3.1.2 Centrality estimation of AP in male and female students

**Figure 2:**
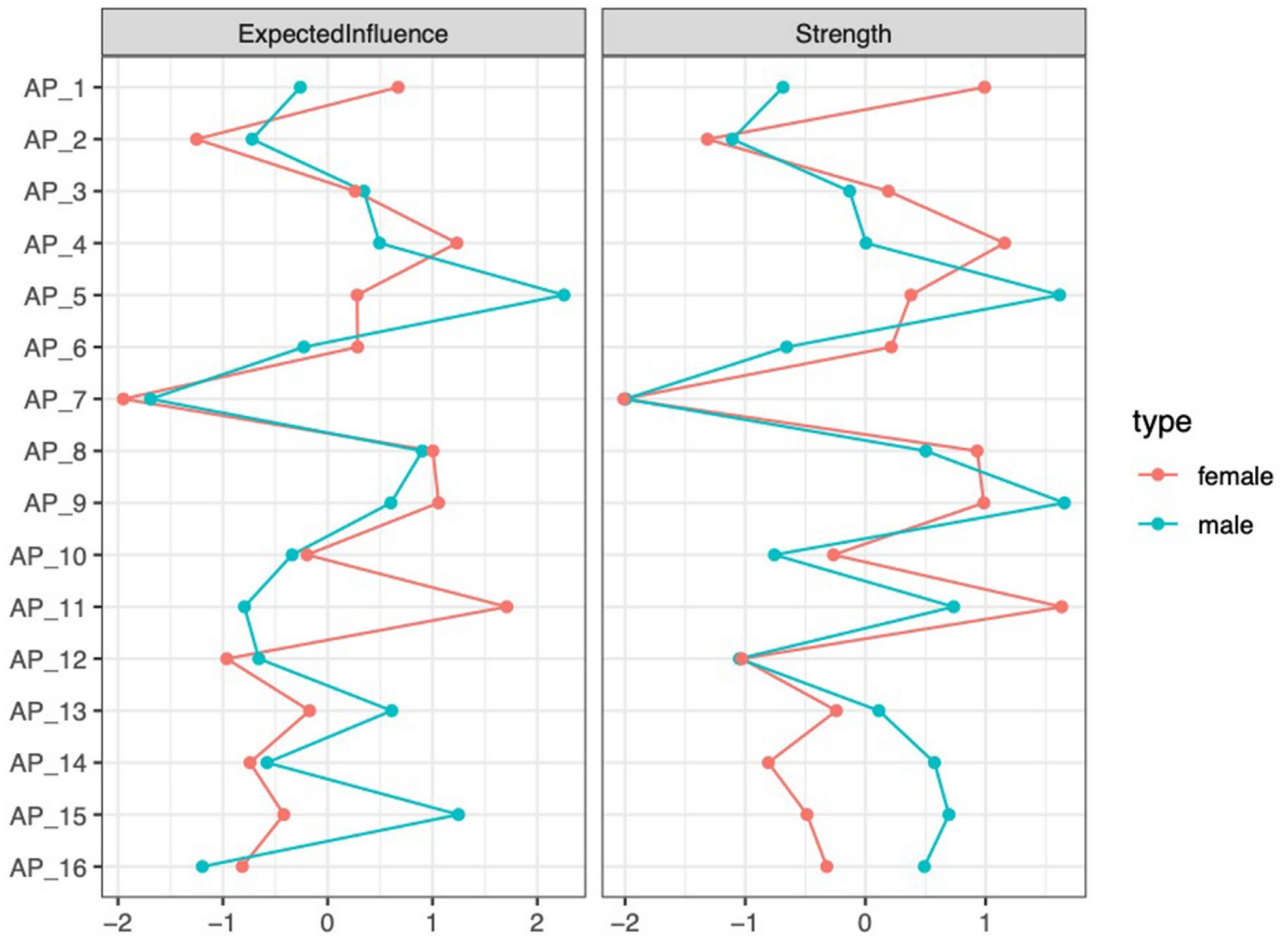
Standardized centrality estimates for AP features are shown for both groups, with the red line representing females and the blue line representing male.

Figure 2 below displays the centrality indices of AP features in both groups. Two metrics are reported: *expected influence*, which captures the overall impact of a node, including the direction (positive or negative) of its connections; and *strength*, which reflects the total connectivity of a node, irrespective of direction. According to Song et al. (2023) suggest, this research selected these two metrics because they are relatively stable and can better reflect the core characteristics. For male students, AP5 (Procrastinating on improving work habits) has the highest expected influence and strength. In contrast, for female students, AP11 (Procrastinating self−promised tasks) shows the highest values for both strength and expected influence.

#### 3.1.3 Network accuracy and stability of AP in male and female students

The accuracy of the networks for both groups was moderately confirmed by the edge weight bootstrapping results (see Appendix Figure A1). For the male group, the centrality indices were 0.206 for strength and expected influence, as indicated by the CS coefficients (see Appendix Figure A2). In contrast, the female group exhibited centrality indices of 0.36 for strength 0.44 for expected influence.

#### 3.1.4 Network comparison of AP in male and female students

Three types of network comparisons were performed between male and female groups. The first, a network structure invariance test, indicated no significant difference in network structure between the groups (*p_male-female_ = 0.769*), suggesting structural similarity. The second test, focusing on global strength invariance, revealed a significant difference in the strength of SA and AP networks between males and females (male = 7.90, female = 7.28; *p_male-female_* = 0.078). Lastly, the edge invariance test identified 33 edges that significantly differed between the two groups. For instance, the connection between AP6 (Making excuses to avoid tasks) and SA4 (Smartphone is indispensable) was notably stronger in males (*p* = 0.006). Further details can be found in Appendix Table A1.

### 3.2 Network of SA among male and female students

#### 3.2.1 Network estimation of SA among male and female students

**Figure 3:**
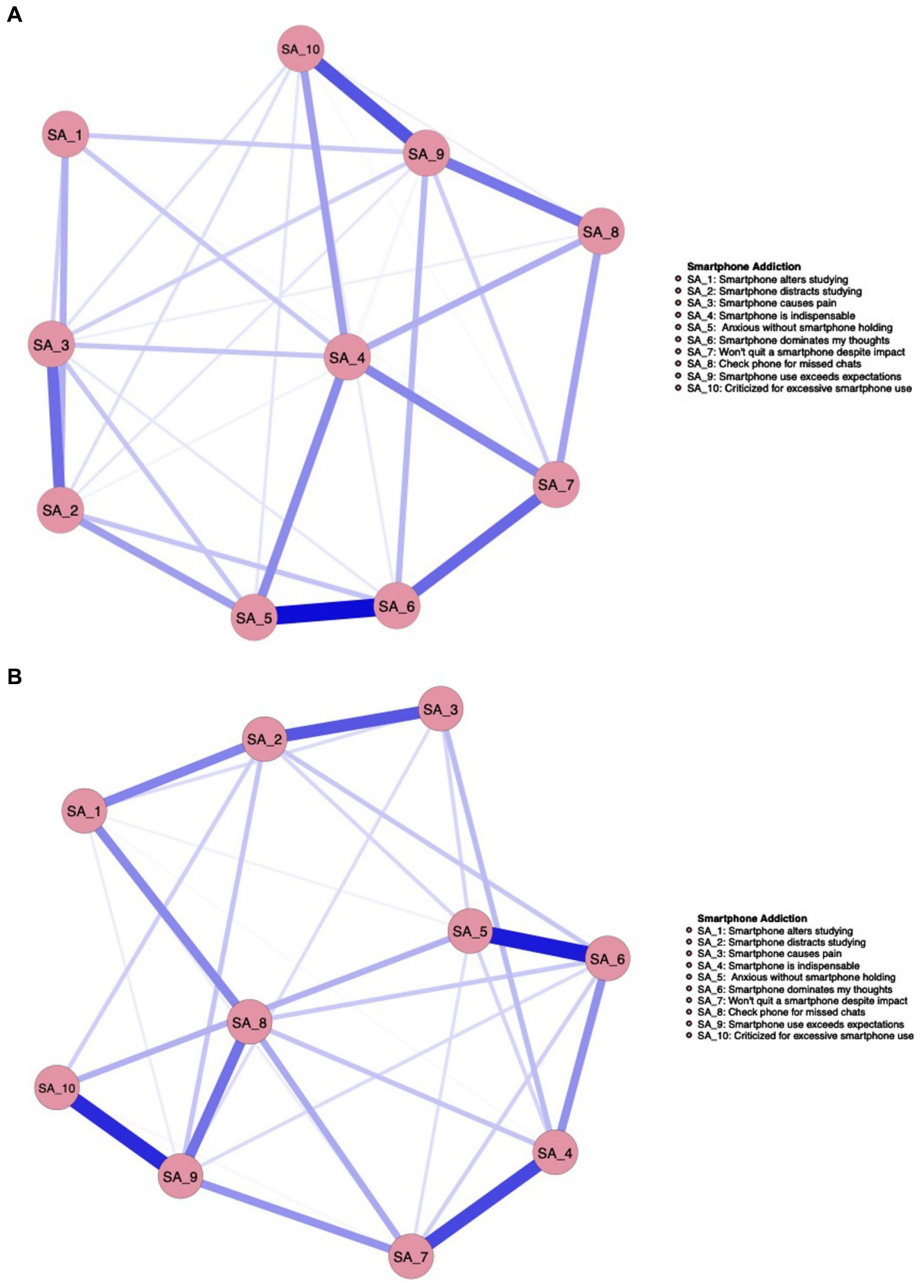
**(A)** Male. **(B)** Female.

The two networks produced a total of 45 edges (10*(10–1)/2), with 34 nonzero-weighted edges in males and 31 in females, as illustrated in Figures 3A,B.

In the visualized networks in Figures 3A,B, nodes correspond to specific SA features. For a detailed explanation of the SA items, see Table 2.

**Table 2:** The network nodes of SA.

Features cluster		Item	Male sample (*N* = 170)		Female sample (*N* = 268)	
			*M*	*SD*	*M*	*SD*
SA 1	*Smartphone addictions*	Smartphone alters studying.	3.08	1.10	2.99	0.96
SA 2	*Smartphone addictions*	Smartphone distracts studying.	3.39	1.06	3.35	1.02
SA 3	*Smartphone addictions*	Smartphone causes pain.	3.43	1.12	3.33	0.99
SA 4	*Smartphone addictions*	Smartphone is indispensable.	3.29	1.26	3.00	1.12
SA 5	*Smartphone addictions*	Anxious without smartphone holding.	3.59	1.14	3.50	0.99
SA 6	*Smartphone addictions*	Smartphone dominates my thoughts.	3.52	1.14	3.41	0.99
SA 7	*Smartphone addictions*	Won’t quit a smartphone despite impact.	3.20	1.19	3.09	1.01
SA 8	*Smartphone addictions*	Check phone for missed chats.	3.27	1.18	3.10	0.99
SA 9	*Smartphone addictions*	Smartphone use exceeds expectations.	3.28	1.18	3.06	1.04
SA 10	*Smartphone addictions*	Criticized for excessive smartphone use.	3.48	1.13	3.40	1.02

#### 3.2.2 Centrality estimation of SA in male and female students

**Figure 4:**
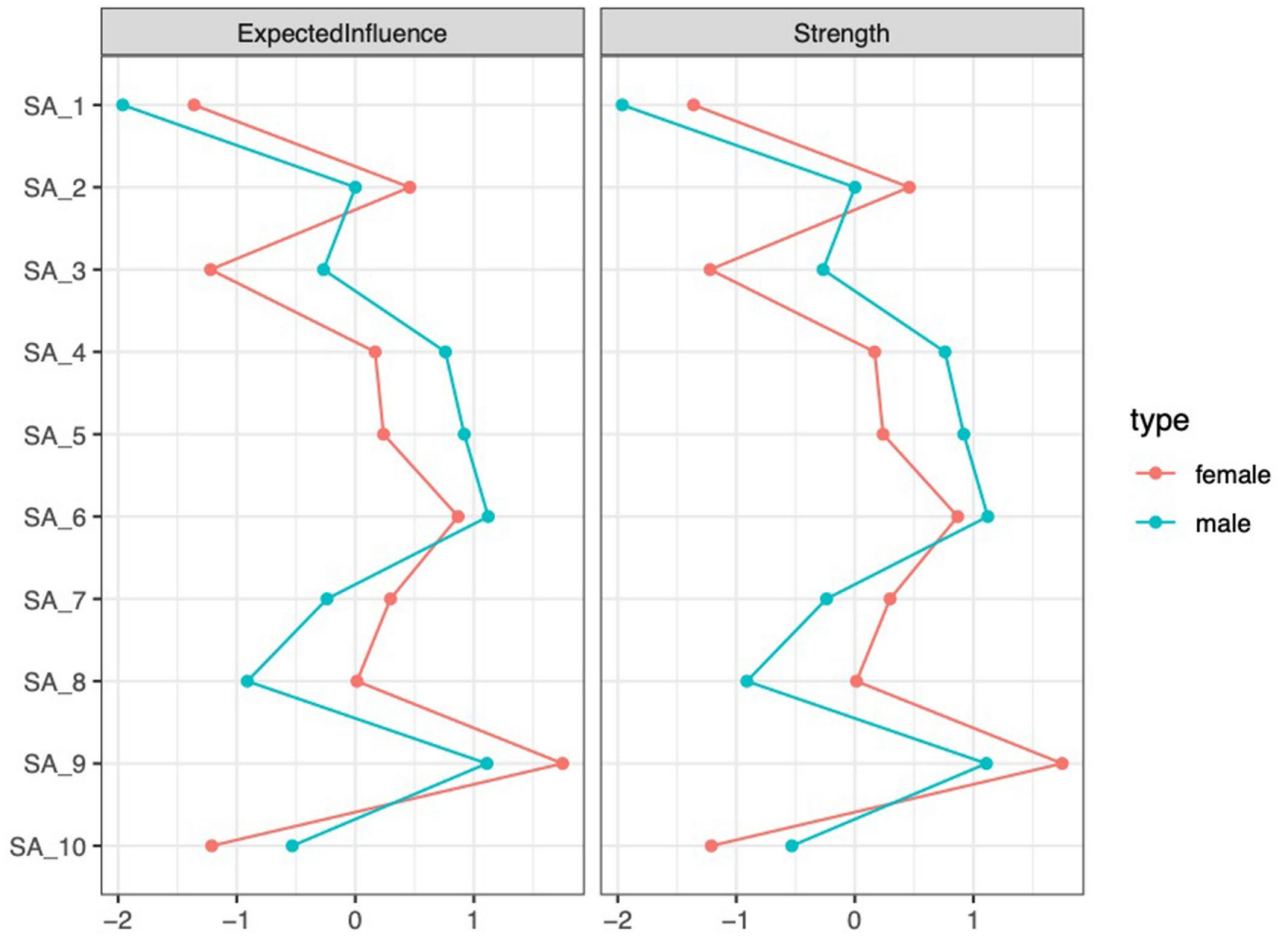
Standardized centrality estimates for SA features are shown for both groups.

Figure 4 below displays the centrality indices of SA features in both groups. For male students, SA6 (Smartphone dominates my thoughts) has the highest expected influence and strength. In contrast, for female students, SA9 (Smartphone use exceeds expectations) shows the highest values for both strength and expected influence.

#### 3.2.3 Network accuracy and stability of SA in male and female students

The accuracy of the SA networks in both groups was moderately supported by the edge weight bootstrapping results (see Appendix Figure B1). For the male group, the CS coefficients indicated centrality indices of 0.359 for both strength and expected influence (see Appendix Figure B2). In comparison, the female group demonstrated centrality indices of 0.44 for strength and 0.515 for expected influence.

#### 3.2.4 Network comparison of SA in male and female students

The network structure invariance test indicated no significant difference in network structure between the groups (pmale-female = 0.625), suggesting structural similarity. The global strength invariance test revealed no significant difference in the strength of SA networks between males and females (male = 4.38, female = 4.43; pmale-female = 0.770). The edge invariance test identified 3 edges that significantly differed between the two groups. Further details can be found in Appendix Table B1.

### 3.3 Interaction network of SA-AP among male and female students

#### 3.3.1 Network estimation of SA-AP among male and female students

**Figure 5:**
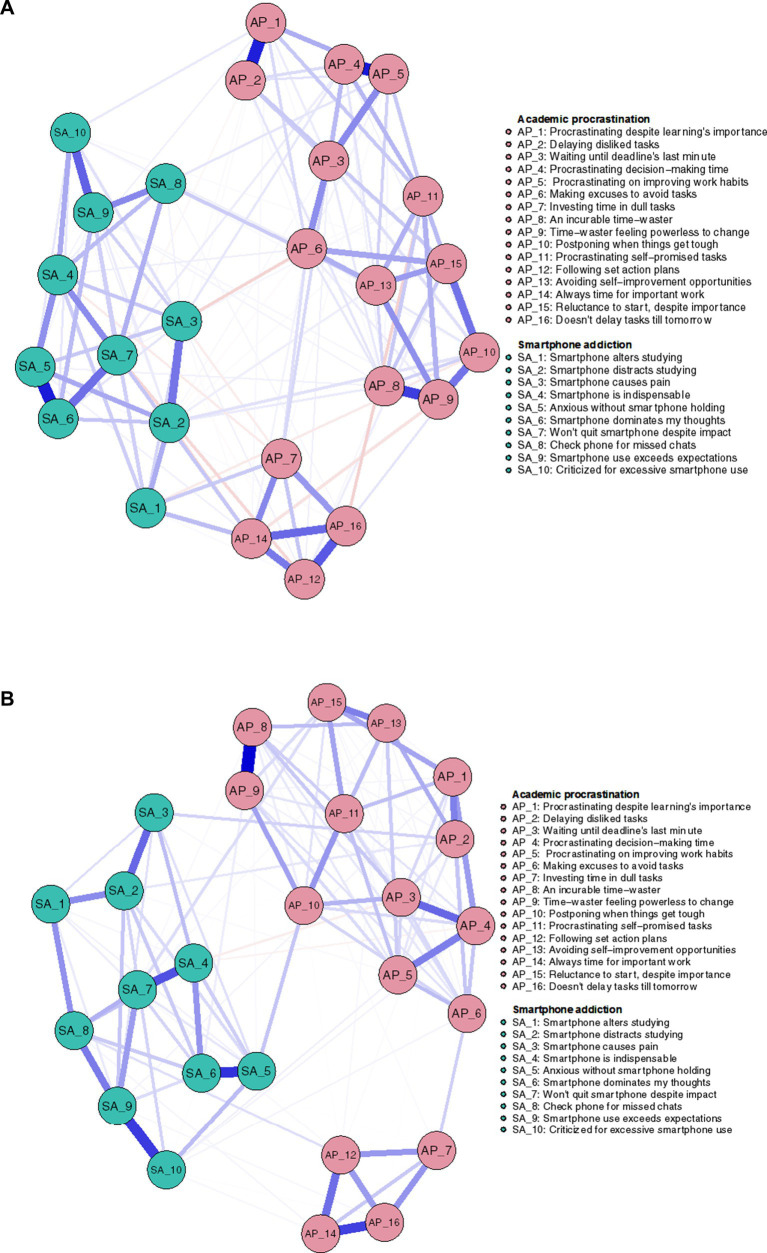
**(A)** Male. **(B)** Female.

The two networks produced a total of 325 edges (26*(26–1)/2), with 135 nonzero-weighted edges in males and 129 in females, as illustrated in Figures 5A,B.

In the visualized networks in Figures 5A,B, nodes correspond to specific SA-AP features.

#### 3.3.2 Centrality estimation of SA-AP in male and female students

**Figure 6:**
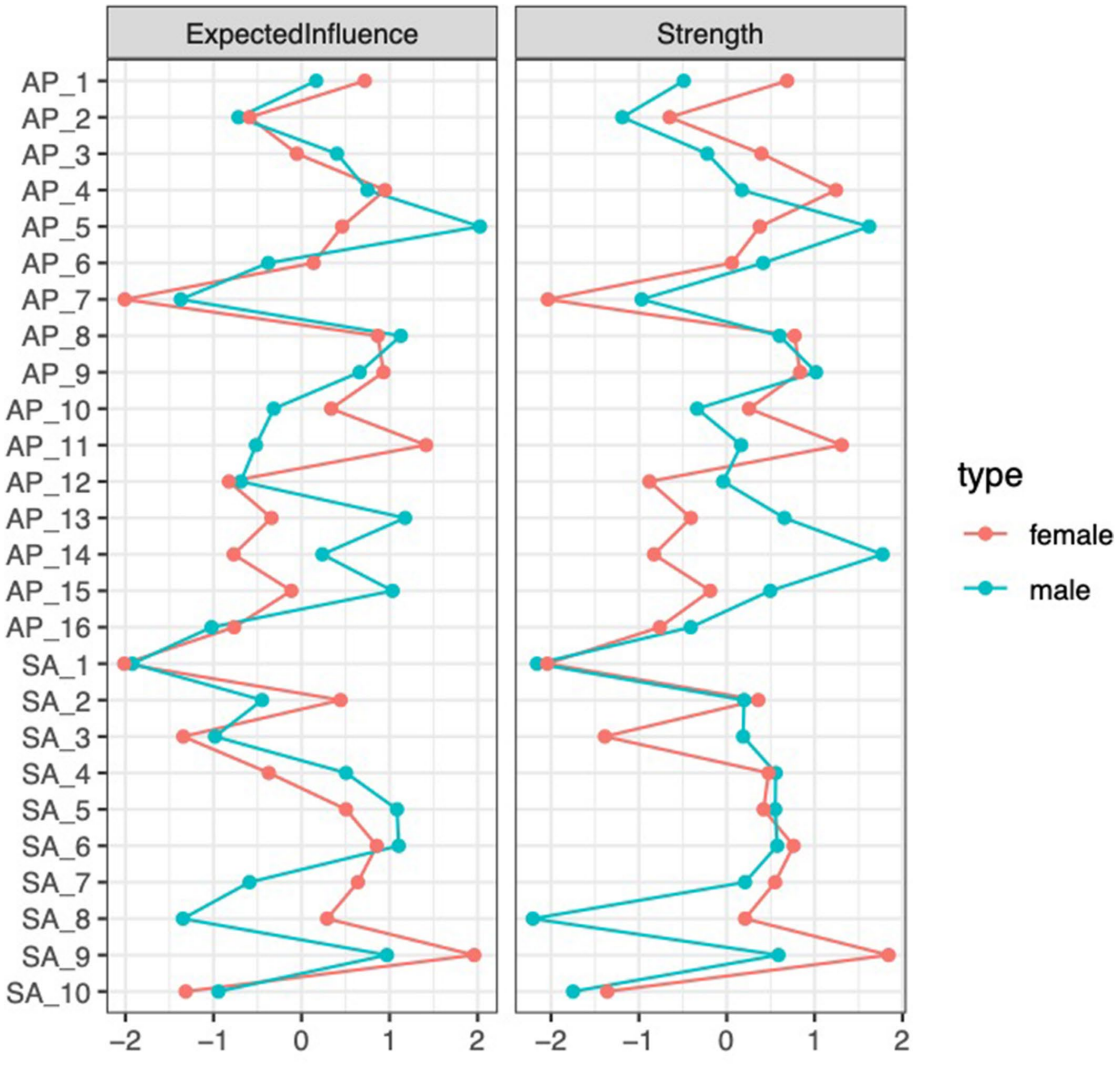
Standardized centrality estimates for SA-AP features are shown for both groups.

Figure 6 below displays the centrality indices of SA-AP features in both groups. For male students, AP5 (Procrastinating on improving work habits) has the highest expected influence, while AP14 (Always time for important work) shows the highest strength centrality. In contrast, for female students, SA9 (Smartphone use exceeds expectations) shows the highest values for both strength and expected influence.

#### 3.3.3 Network accuracy and stability of SA-AP in male and female students

The accuracy of the networks for both groups was moderately confirmed by the edge weight bootstrapping results (see Appendix Figure C1). For the male group, the centrality indices were 0.206 for strength and 0.359 for expected influence, as indicated by the CS coefficients (see Appendix Figure C2). In contrast, the female group exhibited centrality indices of 0.515 for both strength and expected influence.

#### 3.3.4 Network comparison of SA-AP in male and female students

The network structure invariance test indicated no significant difference in network structure between the groups (*p*_male-female_ = 0.856), suggesting structural similarity. The global strength invariance test revealed a significant difference in the strength of SA-AP networks between males and females (male = 13.15, female = 11.97; *p*_male-female_ = 0.005). The edge invariance test identified 33 edges that significantly differed between the two groups. Further details can be found in Appendix Table C1.

## 4 Discussion

Guided by the three research questions, this study employed network analysis to construct network models, identify core features, and conduct network comparisons. The following sections discuss gender differences in the AP network, the SA network, and the SA-AP network.

### 4.1 Gender differences in the AP networks of male and female students

The AP networks of male and female students exhibited more differences than similarities. On the one hand, differences were observed in the core features. For male students, the core feature was AP5 (Procrastinating on improving work habits). According to the delay of gratification theory (Rodriguez et al., 1989), individuals with insufficient self-regulation abilities are more likely to procrastinate when facing long-term goals that require sustained effort. Previous studies have shown that males tend to have weaker self-regulation abilities (Liu et al., 2021; Santamaría-Vázquez et al., 2021). As a result, when confronted with tasks such as improving study habits, which demand long-term commitment, they are more prone to procrastination. In contrast, the core feature for female students was AP11 (Procrastinating self-promised tasks). Females are generally more sensitive to self-promised tasks; however, this sensitivity, coupled with their high pursuit of perfectionistic standards, increases the difficulty of task initiation. As indicated by Pákozdy et al. (2023), females tend to score higher than males on perfectionism, particularly in the dimension of maladaptive perfectionism. On the other hand, no significant differences were observed in the network structure and global strength between males and females, although some differences emerged in the edge strengths of specific connections. This finding addresses Research Question 1 by confirming that, while there are substantial differences in the AP networks between male and female students, some similarities also exist.

### 4.2 Gender differences in the SA networks of male and female students

The SA networks of male and female students also exhibited more differences than similarities. On the one hand, differences were observed in the core features. For male students, the core feature was SA6 (Smartphone dominates my thoughts). Smartphones, as tools for entertainment and gaming, provide males with instant pleasure. Prolonged immersion in such experiences leads to the smartphone content continuously occupying their cognitive space. Even when not using the smartphone, its mere presence captures their cognitive resources, making it difficult for them to concentrate on academic tasks (Ward et al., 2017). In contrast, the core feature for female students was SA9 (Smartphone use exceeds expectations). Females tend to use smartphones more as a medium for social interaction and relationship maintenance (Gezgin, 2021). In order to sustain their social connections and avoid neglecting them, they often need to constantly monitor smartphone updates. This immediate social feedback makes it difficult for them to control the duration of their smartphone use, resulting in usage that exceeds their expectations. On the other hand, no significant differences were observed in the network structure and global strength between males and females, although some differences emerged in the edge strengths of specific connections. These findings address Research Question 2 by indicating that, while there are distinct differences between males and females, certain similarities also exist, which differs from the conclusions of previous research.

### 4.3 Gender differences in the SA-AP interaction network among male and female students

In the network formed by the interaction between SA-AP, differences also outweigh similarities. Specifically, discrepancies are observed in core features, global strength, and edge strength, whereas the network structure remains similar. These findings address Research Question 3.

#### 4.3.1 Core features and gender differences of SA and AP

The results of the analysis suggest that AP5 (Procrastinating on improving work habits) and AP14 (Always time for important work) are the core features of the male network, whereas SA9 (Smartphone use exceeds expectations) is identified as the core feature in the female network. The findings of this study in male students align with Üztemur and Dinç's (2023) proposition that males exhibit stronger AP compared to females. The prominence of AP5 in males may be explained by gender stereotypes in academic environments (Schunk and Pajares, 2002). According to Social Role Theory (Eagly and Wood, 2012), societal expectations assign distinct roles to males and females, shaping their behaviors through socialization. In academic settings, males may perceive learning tasks as incompatible with masculine norms, leading them to procrastinate as a way to align with their perceived gender role (Üztemur and Dinç, 2023). The identified core feature of AP14 (Always time for important work) suggests that male students perceive they can complete academic tasks with time to spare. This may not be the case, as this finding contradicts the assertions of Košíková et al. (2020) and Balkis and Duru (2017). They argue that male students generally have poor time management skills, which is a significant factor contributing to AP. Klassen (2007) proposes that males tend to misjudge and overestimate their abilities. This could also justify why AP14 (Always time for important work) is identified as one of the core features of male students, as they could misjudge and overestimate their ability to complete academic tasks by believing they have ample time.

On the other hand, the identified core feature in female students is SA9 (smartphone use exceeds expectations). This finding is consistent with the findings of Aparicio-Martínez et al. (2020) and Zhao et al. (2021), they suggest that females exhibit greater SA compared to males. One possible explanation for this result is triggered by academic stress (Tu et al., 2023). When encountering stressful life incidents, such as academic pressure or challenging academic tasks, females tend to seek social support and emotional relief through social interaction (Gentry et al., 2007; Tifferet, 2020). This tendency stems from their high need for interpersonal relationships (Kendler et al., 2001; Verma et al., 2011), and smartphones are undoubtedly the most accessible and convenient tools that enable social networking to satisfy this need for females. This thus justifies why females exhibit higher levels of addiction to smartphone-related behaviors, such as social media usage than males when under academic pressure (Tu et al., 2023). However, excessive reliance on smartphones for social interactions may also lead females to exceed their intended use.

The findings show that AP is the core feature for male students, while SA is central for female students. This difference stems from distinct coping styles under academic pressure. Shih (2017) highlights that Asian student, especially in China, face intense academic pressure due to high family and societal expectations. In collectivist cultures, students often prioritize these expectations over personal goals (Özer et al., 2009), making academic success a key measure of self-worth (Ang and Huan, 2006). Males, in particular, tend to adopt avoidance strategies under stress, such as procrastination and excessive smartphone use (Üztemur and Dinç, 2023). Eschenbeck et al. (2007) further explain that males cope with stress through emotional suppression and avoidance, which may explain why AP emerges as their core feature. In contrast, female students are more likely to cope by seeking social support and emotional relief through social interaction (Gentry et al., 2007; Tifferet, 2020). When facing negative emotions, they tend to prefer activities that offer immediate rewards and feedback (Cho et al., 2017), with smartphones providing an accessible outlet (Yang et al., 2020). This makes females more vulnerable to excessive smartphone use under academic pressure (Aparicio-Martínez et al., 2020; Zhao et al., 2021).

#### 4.3.2 Gender differences between SA and AP networks structure

The study results suggest that the network structure of SA and AP is similar between the two groups, indicating that the nodes in the networks of males and females are connected in a similar way. This implies comparable interactions between the features in the female and male groups, but there are differences in terms of global strength and edge strength. One hand, The global strength of males is found to be stronger than that of females, indicating that the feedback loops between features of the SA and AP networks are stronger in males compared to females. This finding aligns with Üztemur and Dinç’s (2023) assertions and Yang et al. (2020) who suggest that male students tend to experience higher academic pressure and are more inclined to access the internet and utilize it more frequently to ease such pressure. Moreover, in collectivist cultures, males typically have less strict discipline and thus have more access to smartphones. On the other hand, differences also exist in the edge strength of the SA and AP networks between males and females. For example, the association between AP6 (Making excuses to avoid tasks) and SA4 (Smartphone is indispensable) is stronger in males than in females. Eschenbeck et al. (2007) contend that males are more inclined to adopt avoidance strategies to cope with academic pressure, and games and short videos on smartphones are very appealing to them (Tu et al., 2023). Therefore, smartphones become indispensable tools for avoiding academic tasks.

## 5 Implication

This study innovatively employed network analysis to examine gender differences in the AP, SA, and SA-AP interaction networks, providing new theoretical perspectives and empirical evidence for this field. Unlike traditional studies that primarily focus on mean-level differences between male and female students, network analysis enables an in-depth exploration of the internal characteristics and structural differences of the variables. This methodological advancement contributes to findings that differ from previous research, further enriching the literature on gender differences in AP and SA.

While prior research on gender differences has largely focused on determining which gender experiences more severe levels of AP and SA, the results of this study suggest that gender differences extend beyond severity. More importantly, males and females exhibit distinct psychological characteristics, behavioral patterns, and interaction mechanisms related to AP and SA. Specifically, for male students, the core feature is procrastination related to improving study habits, reflecting challenges in self-regulation and managing long-term goals. In contrast, the core feature for female students is excessive smartphone use, indicating a strong reliance on immediate social feedback and the consequent risk of losing control over smartphone usage. These differences imply that gender not only influences the manifestation of problematic behaviors but also profoundly shapes the underlying psychological motivations.

These findings offer valuable insights for psychological education and behavioral interventions in higher education settings. First, for male students, interventions should focus on enhancing self-regulation and time management skills to help them recognize and overcome procrastination patterns in developing effective study habits. Second, for female students, interventions should aim to manage their reliance on instant feedback, promote healthier social behavior patterns, and strengthen emotional regulation strategies to reduce excessive smartphone use driven by social anxiety. Finally, when designing intervention programs, it is essential to recognize that male and female students respond differently to similar academic pressures, and gender-specific strategies are necessary to effectively mitigate AP and SA problems among university students.

## 6 Limitations and future study

Like many other research studies, this study has certain limitations. Given that the participants of the study are focused on universities in China, the generalizability of the findings is only applicable to Chinese students. Therefore, future studies could explore gender differences in SA and AP, taking into consideration the impact of societal cultures and comparing across different cultures. Additionally, the literature suggests that SA could be further divided into social media addiction, gaming addiction, and short video addiction. According to Tu et al. (2023), significant differences exist between males and females in these three types of addictive behaviors. Therefore, future researchers could probe more thoroughly into the relationship between different types of SA and AP, as well as gender differences.

## 7 Conclusion

This study employed network analysis to comprehensively explore gender differences in the AP network, the SA network, and the SA-AP interaction network among university students. The findings revealed that there were more differences than similarities between male and female students in the SA and AP networks. In the SA-AP interaction network, male students were primarily characterized by procrastination behaviors and delays in improving study habits, reflecting deficiencies in self-regulation and long-term goal management. In contrast, female students exhibited a strong reliance on immediate social feedback, which led to uncontrolled smartphone use. These findings suggest that gender not only influences the severity of problematic behaviors but also profoundly shapes the underlying psychological motivations and behavioral mechanisms. The study provides valuable insights for the development of targeted psychological education and behavioral interventions in higher education. Future research should further investigate gendered behavioral patterns through longitudinal studies and cross-cultural validation, thereby enhancing the precision and effectiveness of mental health initiatives in universities.

## Data Availability

The original contributions presented in the study are included in the article/Supplementary material, further inquiries can be directed to the corresponding author/s.

## Footnotes

^1^ https://www.wjx.cn/
