# Supplementary material for: Mapping gender networks of smartphone addiction and academic procrastination: a network analysis study
Source: Front Psychol. 2025 Jun 18;16:1557684. doi: 10.3389/fpsyg.2025.1557684 (PMC12213621; doi:10.3389/fpsyg.2025.1557684)
Supplement: Supplementary file 1 [file Supplementary_file_1.pdf]

Appendices

Appendix Fig. A1 indicate supplementary figures in edge weight accuracy of AP in male and female students.

Appendix Fig. A2 indicate supplementary figures in centrality stability of AP in male and female students.

Appendix Table. A1 indicate supplementary data in edge invariance test between two networks.

Appendix Fig. B1 indicate supplementary figures in edge weight accuracy of SA in male and female students.

Appendix Fig. B2 indicate supplementary figures in centrality stability of SA in male and female students.

Appendix Table. B1 indicate supplementary data in edge invariance test between two networks.

Appendix Fig. C1 indicate supplementary figures in edge weight accuracy of SA-AP in male and female students.

Appendix Fig. C2 indicate supplementary figures in centrality stability of SA-AP in male and female students.

Appendix Table. C1 indicate supplementary data in edge invariance test between two networks.

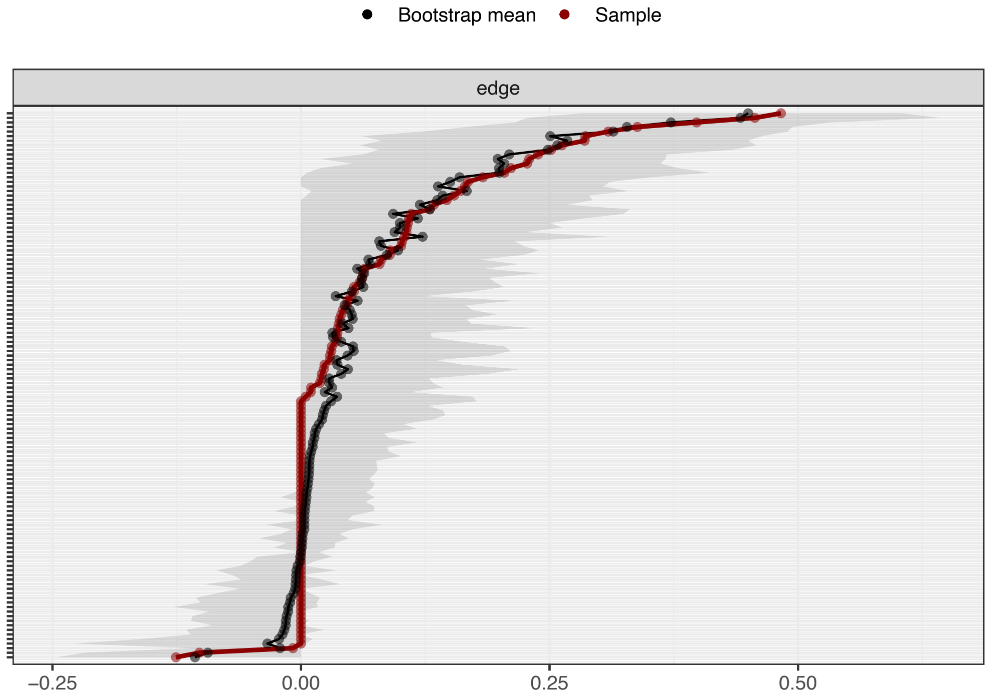

A. Male

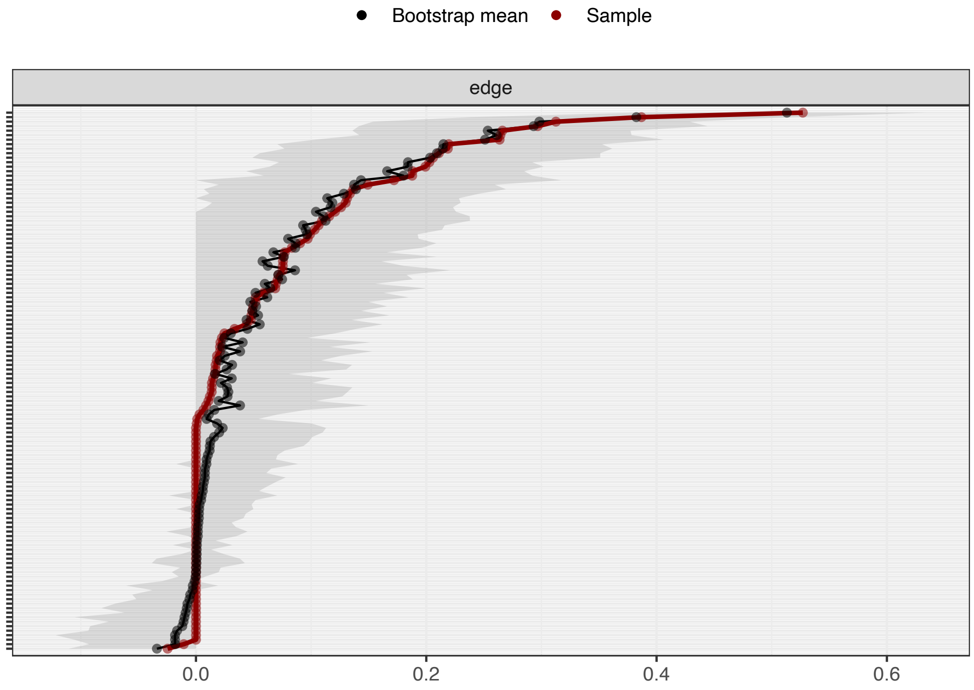

B.Female

Appendix Fig. A1 Edge weight accuracy for AP feature network in male and female. The red line represents the sample value. The gray is indicates the 95% confidence intervals. Each horizontal line represents one edge of the network, ordered from the edge with the highest edge-weight to the edge with the lowest edge weight.

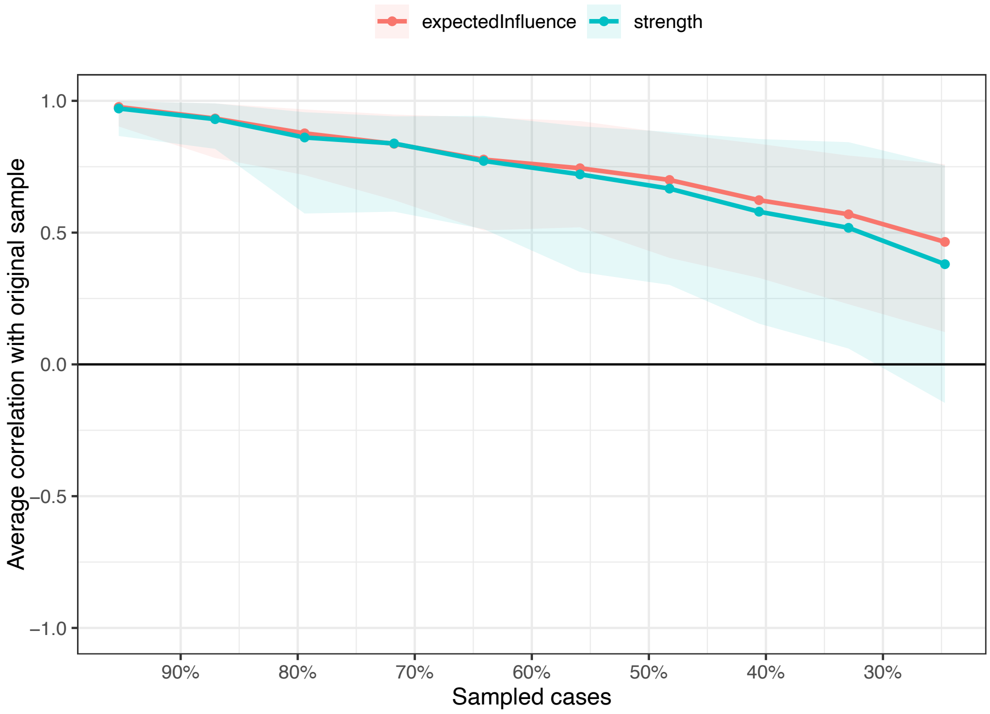

A.Male

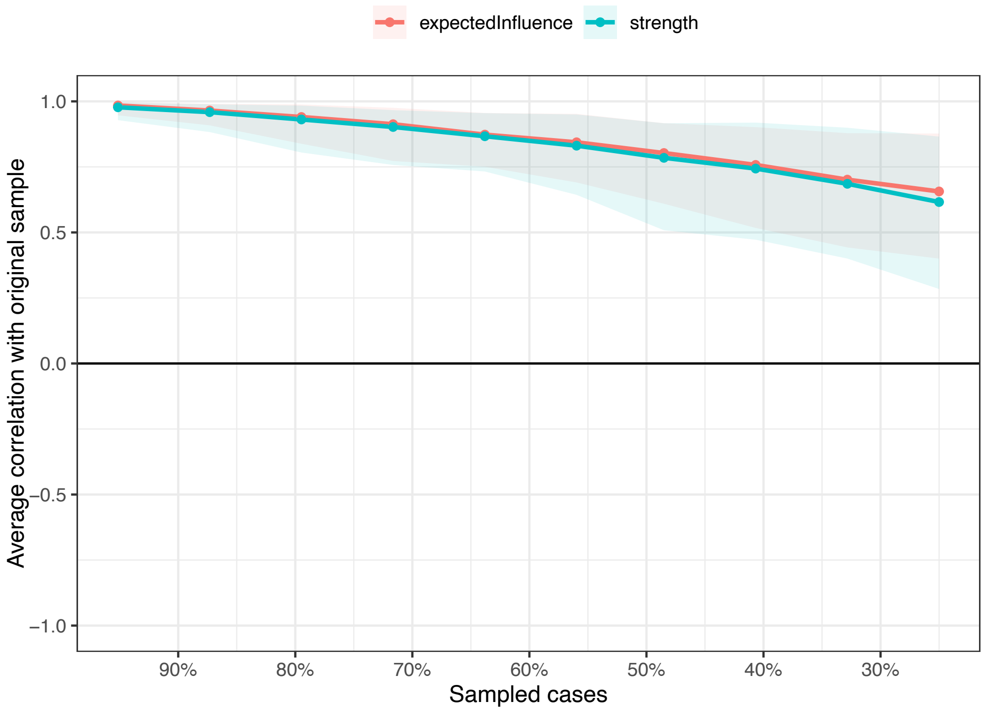

B.Female

Appendix Fig. A2 Centrality stability for AP features network. The x-axis illustrates the sample decrease from 95% to 25% of the original sample, and the y-axis illustrates the changes in correlation estimates between the subsample and the original entire sample. Lines indicate the means, and areas indicate the range from the 2.5th quantile to the 97.5th quantile.

Appendix Table A. 3 Edge invariance test between male and female(P<0.05)

Edge between		*P*-value
AP_10	AP_16	0.046
AP_4	AP_13	0.045
AP_1	AP_15	0.045
AP_10	AP_11	0.038
AP_4	AP_5	0.037
AP_9	AP_14	0.034
AP_8	AP_15	0.025
AP_11	AP_14	0.018
AP_1	AP_4	0.016
AP_9	AP_13	0.012
AP_3	AP_7	0.007
AP_11	AP_16	0.005

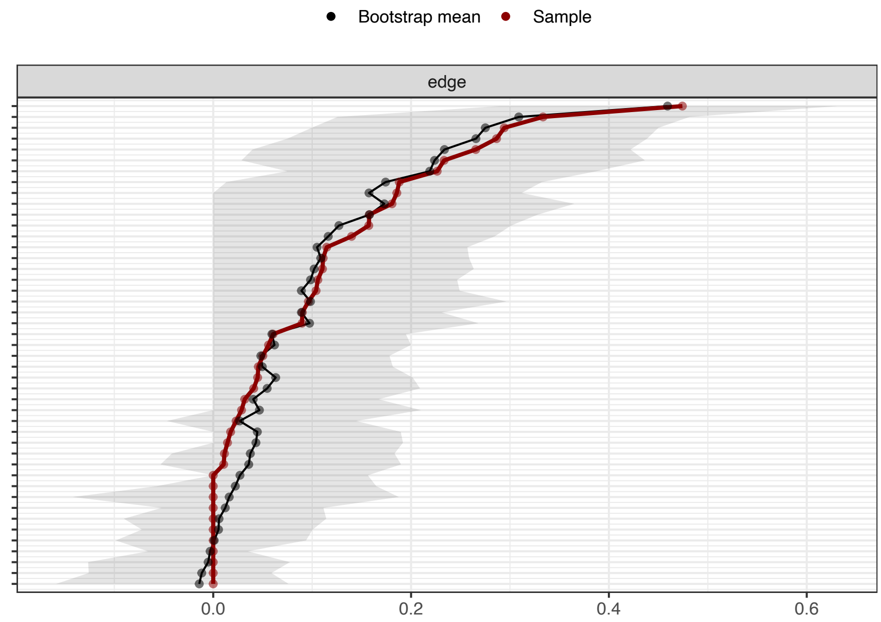

A,Male

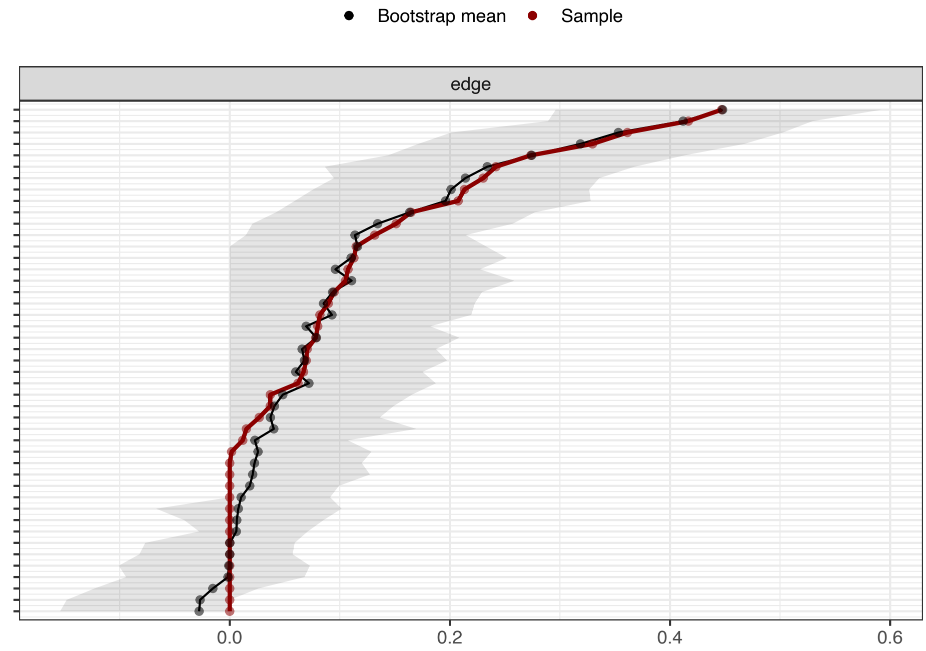

B.Female

Appendix Fig. B1 Edge weight accuracy for SA feature network in male and female. The red line represents the sample value. The gray is indicates the 95% confidence intervals. Each horizontal line represents one edge of the network, ordered from the edge with the highest edge-weight to the edge with the lowest edge weight.

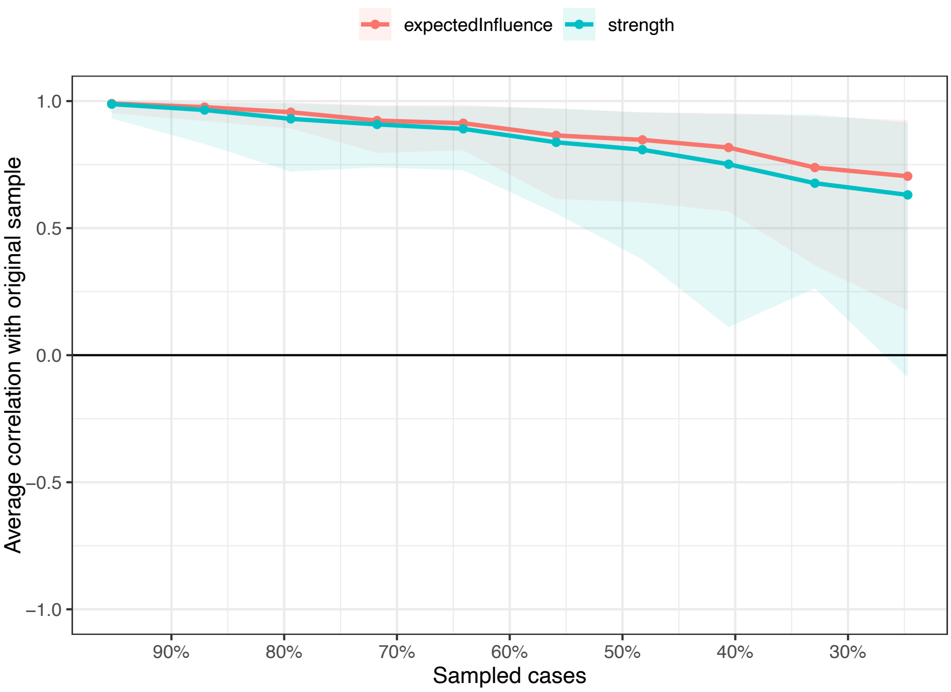

A.Male

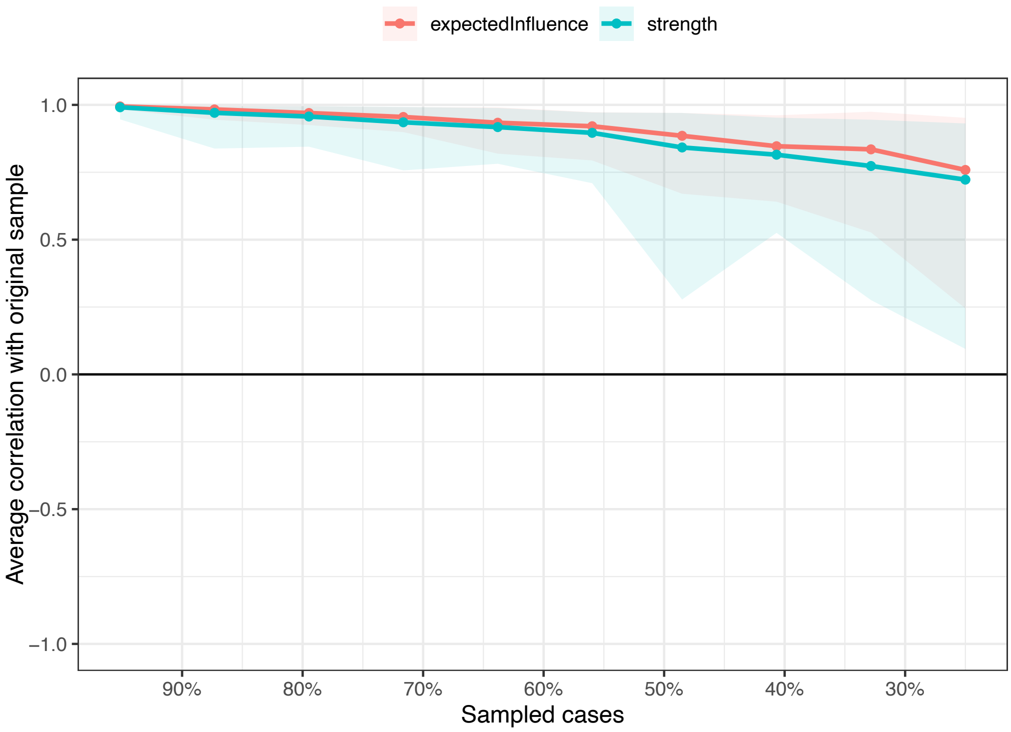

B.Female

Appendix Fig. B2 Centrality stability for SA features network. The x-axis illustrates the sample decrease from 95% to 25% of the original sample, and the y-axis illustrates the changes in correlation estimates between the subsample and the original entire sample. Lines indicate the means, and areas indicate the range from the 2.5th quantile to the 97.5th quantile.

Appendix Table B. 1 Edge invariance test between male and female($P<0.05$)

Edge between		*P*-value
SA_4	SA_6	0.038
SA_1	SA_8	0.031
SA_4	SA_10	0.004

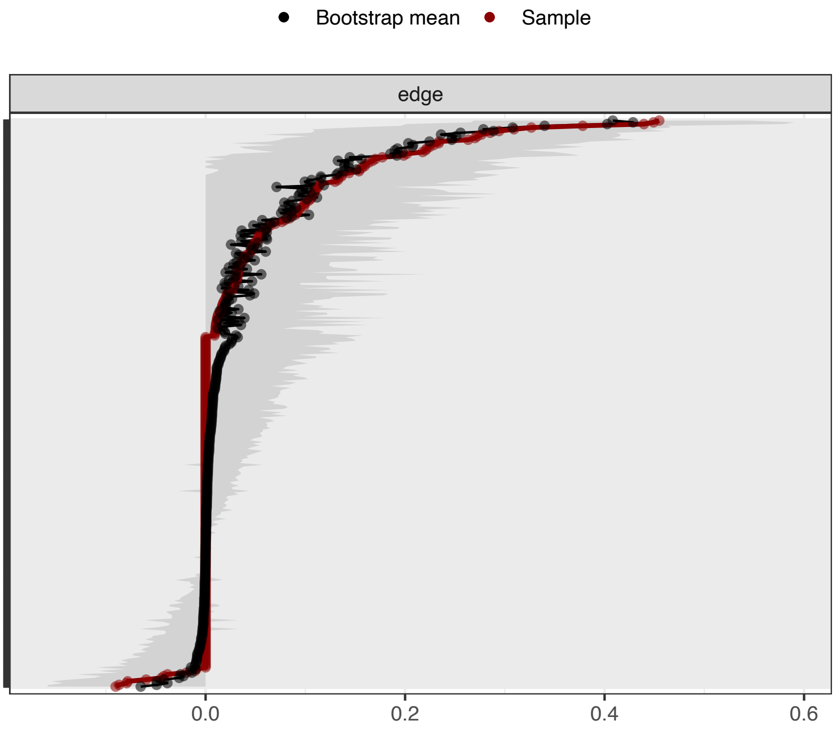

A. Male

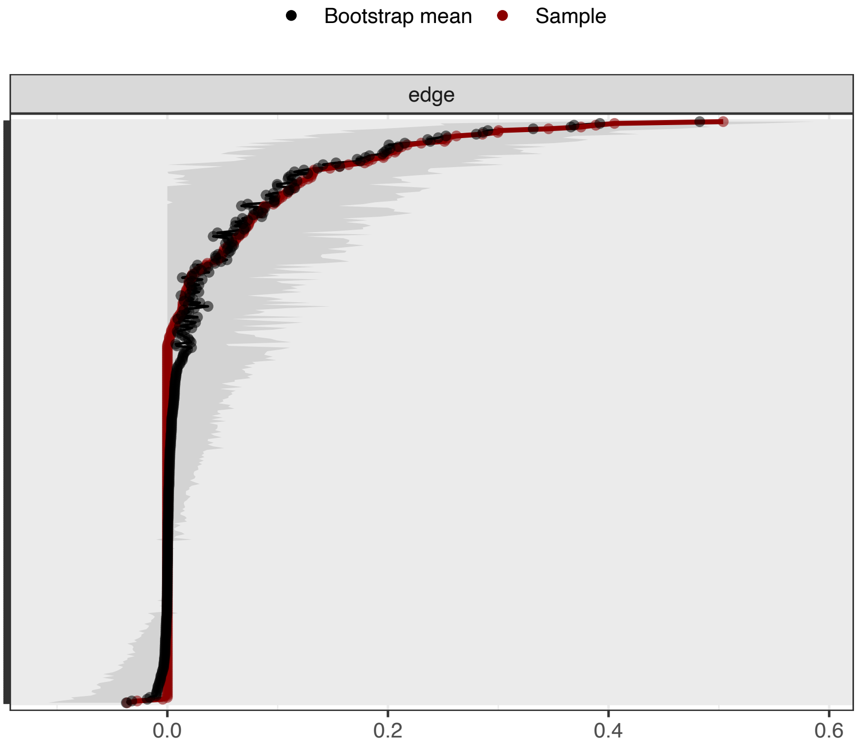

B. Female

Appendix Fig. C1 Edge weight accuracy for SA and AP feature network in male and female. The red line represents the sample value. The gray is indicates the 95% confidence intervals. Each horizontal line represents one edge of the network, ordered from the edge with the highest edge-weight to the edge with the lowest edge weight.

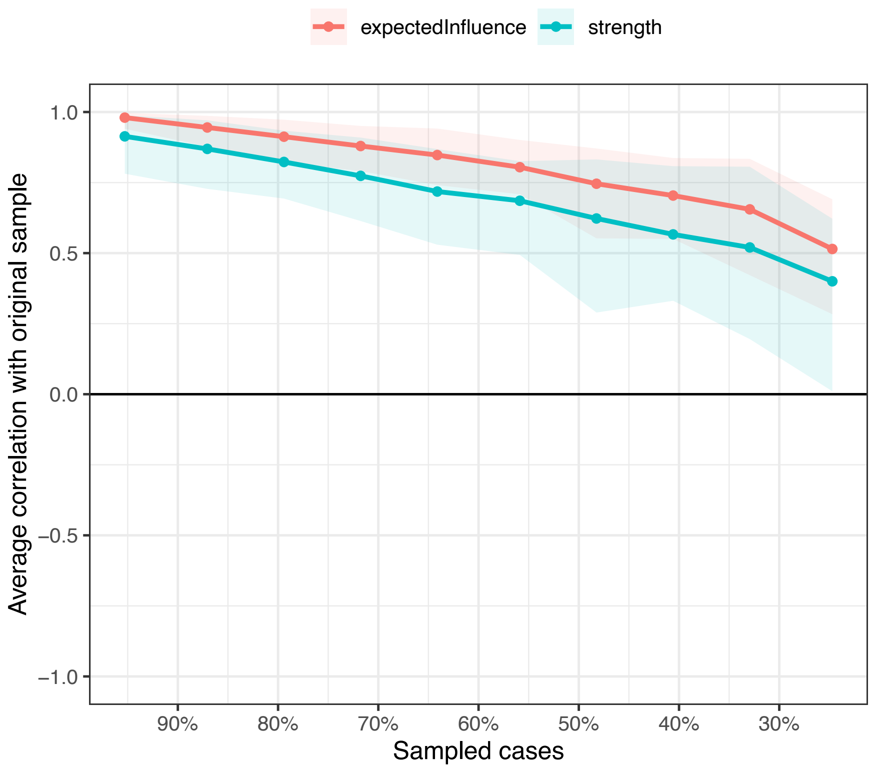

A. Male

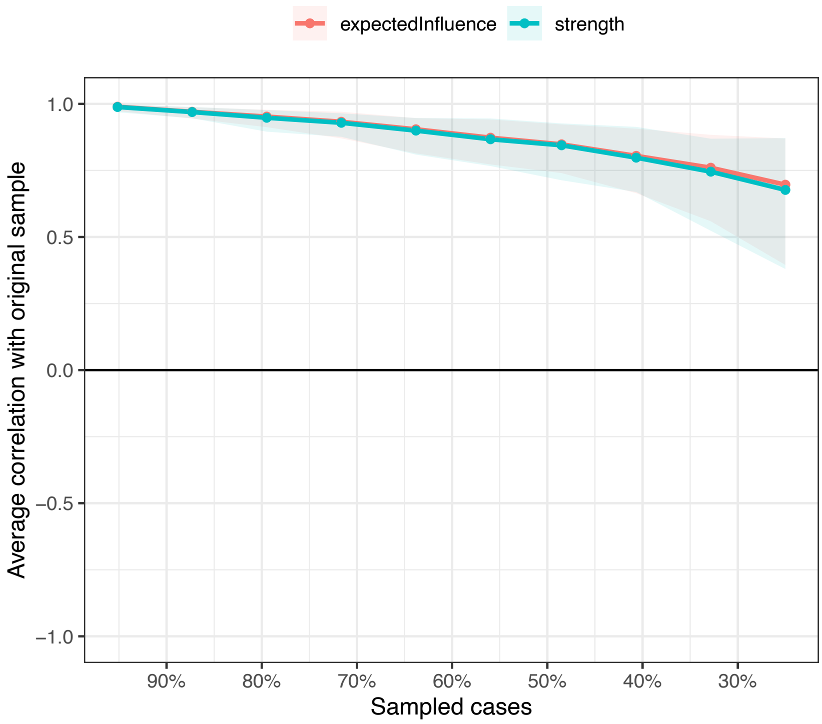

B. Female

Appendix Fig. C2 Centrality stability for SA and AP features network. The x-axis illustrates the sample decrease from 95% to 25% of the original sample, and the y-axis illustrates the changes in correlation estimates between the subsample and the original entire sample. Lines indicate the means, and areas indicate the range from the 2.5th quantile to the 97.5th quantile.

Appendix Table C.1 Edge invariance test between male and female(P<0.05)

Edge between		P-value
AP_12	SA_9	0.047
AP_1	AP_15	0.045
AP_4	AP_13	0.044
AP_10	SA_5	0.041
SA_4	SA_6	0.038
AP_14	SA_2	0.035
AP_4	AP_5	0.034
AP_10	AP_11	0.032
AP_9	AP_14	0.026
AP_16	SA_5	0.026
AP_7	SA_4	0.023
AP_4	SA_7	0.022
SA_1	SA_8	0.022
AP_8	AP_15	0.019
AP_6	SA_2	0.018
AP_2	SA_7	0.017
AP_12	SA_8	0.016
AP_1	AP_4	0.014
AP_13	SA_8	0.014
AP_9	AP_13	0.012
AP_14	SA_1	0.012
AP_10	SA_1	0.008
AP_11	AP_16	0.007
AP_4	SA_3	0.007
AP_14	SA_7	0.007
AP_6	SA_3	0.006
AP_6	SA_4	0.006
AP_1	SA_4	0.005
SA_4	SA_10	0.005
AP_3	AP_7	0.003
AP_5	SA_8	0.003
AP_10	SA_2	0.002
AP_12	SA_7	0.001
